# Can bioethics bray? Non-human animals, biosemiotics, and a road to shared decision-making

**DOI:** 10.1007/s11017-025-09705-6

**Published:** 2025-02-26

**Authors:** Martin J. Fitzgerald

**Affiliations:** Department of Biomedical Education and Anatomy, Center for Bioethics and Medical Humanities, College of Medicine, The Ohio State University Wexner Medical Center, Columbus, OH USA

**Keywords:** Animal ethics, Shared decision-making, Semiotics, *Umwelt* theory

## Abstract

The prospect of shared decision-making with animals is an elusive one. Its elusiveness comes largely from how difficult it is to assess the linguistic abilities of animals, whether that be their ability to ‘speak’ or their ability to maintain propositional values. In this paper, I suggest a path to shared decision-making with animals that attempts to avoid these deadlocks by using resources from biosemiotics and *Umwelt* theory. I begin with an examination of the general structure of decision-making, demonstrating its future-orientation, comparison of imagined futures, and assessment of what things matter to participants in decision-making. Animals’ capability of having things matter to them, due to their residence in *Umwelten*, offers a means to shared decision-making with animals via a process I call ‘imaginative adjuncting.’

## Introduction

Making just decisions involving non-human animals (hereafter ‘animals’) is no easy matter. Animals do not send representatives to speak on their behalf as kingdoms, species, populations, or packs. Even in those situations where humans address animals one-on-one, it is still notoriously difficult to figure out what to do. Humans are disadvantaged in that they cannot ask animals what they themselves want for all but the most trivial cases. And, even then, humans cannot use their full linguistic capacities to interact with animals. Despite humans’ best efforts thus far, humans have not successfully taught anything but the most rudimentary pieces of human language to animals [1]. What does this mean? The scholarly debate about what animal communication implies about animal language abilities (and whether they are continuous with and/or analogous to human language capabilities) is controversial, to say the least [2]. While it is (relatively) clear that (many)^1^ animals can form concepts of one sort or another [3, ch. 5; 1, p. 292], it is decidedly unclear what to make of the mental lives of animals in light of their conceptual capabilities. For instance, are animals thinking with a kind of language? Is there such a thing as a language of thought? Or, are mental concepts really just predilections to act a certain way? On one hand, there must be some neural reason why human brains are so good at processing language [4]. On the other hand, it is unclear if human capacity for language is the result of innate grammatical structures (e.g. Chomsky’s Universal Grammar) or more domain-general learning abilities [5]. The part of human language animals are weakest at—or at least the part that is supported by empirical data in the weakest way—is syntax [1]. And yet, how does syntax relate to cognition? The cycle continues.

It strikes me that these sorts of questions are in principle solvable. Most of the experiments involving manually signed languages in non-human primates were plagued with methodological errors, including a linguistic chauvinism that had experimenters who did not speak American Sign Language attempting to use manually signed English as a proxy [1, ch. 7]. Irene Pepperberg has carried out much better experiments with Alex the Parrot [6], so one could imagine experimenting with other animals or returning to non-human primates. Then again, one should perhaps be grateful that these experiments are over, as the apes involved by and large did not live pleasant lives [7]. For the time being, though, human-animal communication appears stuck. Animals are worthy of moral respect, but when it comes time to make decisions *with* them, it is unclear how humans should proceed, or whether it is even conceptually possible to proceed. As a practical matter, this means a decision-maker often defers to an animal’s human owner to make a choice. This model is certainly expedient, but it is does not live up to the task of recognizing the moral worth of animals. At best, one could hope that the owner acts benevolently and has the animal’s best interests in mind. At worst, the animal’s interests are erased altogether.

Because humans cannot directly speak with animals, decision-making between humans and animals tends to get trapped between these two poles of benevolent paternalism and outright erasure. If this as an undesirable state of affairs (and I certainly think it to be), then a way out is needed. One option is to wait and hope that animals both are capable of the types of conceptual representation that would allow humans to converse with them, and that science can build ingenious devices or devise ingenious methods that would allow humans to talk to them. This is highly desirable but leaves the problem in stasis in the present.

However, I do not think these possibilities are the only ones. In the bioethics literature, much has been made of the possibility of shared decision-making, even in cases when the patient is profoundly—though not *completely*—incapacitated. Shared decision-making (hereafter ‘SDM’) is a process whereby decision-making involving a patient occurs between the patient, their care team, and others as appropriate (e.g. friends, family, confidants). It is a process in which these participants commit to mutual exchange, mutual earnestness, and mutual engagement. Importantly, it is not a philosophical debate of principle, even though debates of principle may appear in the working-out of SDM.

My central claim in this paper is that animals’ residence in semiotically^2^ meaningful *Umwelten*,^3^ to borrow the term from von Uexküll [8], allows for shared decision-making with animals*.* Although animals may or may not have the linguistic ability to directly engage with SDM, they nevertheless inhabit a meaningful world that matters to them. This provides a tantalizing connection to the structure of SDM, which demands that participants engage in mutual imagining of possible futures with regards what would matter about these futures. From this, SDM with animals becomes possible insofar as they are afforded representatives who can act as imaginative adjuncts, by which I mean someone who can attempt to imagine on behalf of someone else to determine what a given possible future would *mean* and how it would *matter* to that other person. Along the way, I will show that this caveat separates SDM from stakeholder analysis, which attempts to compare not *meanings,* but *values.*

The full expansion of these terms will have to wait until later in the paper. In the meantime, I will note the President’s Commission’s insistence that “ethically valid consent is a process of shared decisionmaking based upon mutual respect and participation, not a ritual to be equated with reciting the contents of a form that details the risks of particular treatments” [9, p.2]. Thus, what results from this analysis is not the contents of the exact procedure of SDM, but rather a demonstration of its possibility. In other words, I attempt to show that SDM with animals is theoretically possible to perform, even if I do not provide the exact means by which to do it. To begin, I now turn to the structure of shared decision-making itself.

## The structure of shared decision-making

SDM is “an *ethos* rather than a mechanised model to be applied in practice” [10].^4^ It is a means by which the process of decision-making and the authority to make decisions is shared among relevant participants rather than simply vested in the authority of either a single doctor or deferred entirely to a patient or some other party. The ultimate goal of SDM is to allow a patient to become an active participant in the medical decision-making process by expressing their own values and preferences. Hence, SDM has been referred to as “the pinnacle of patient-centered care” [11]. SDM is related to supported decision-making, where supported decision-making is more explicitly about partially incapacitated people. Its premise to it is to establish “a process by which people who might otherwise be unable to make their own decisions do so with help from other people” [12, p. 37]. Either way, SDM—and its relatives—press for the inclusion of patients in the decision-making process over and against the temptation of simply deciding on behalf of another. SDM is a process of mutual exchange, whereby participants in the process engage with earnestness, bringing different considerations before one another, deliberating upon them, and attempting to decide together what is to be done.

## Imagination and the temporality of decisions

At the root of shared decision-making is decision-making itself, which is usually studied under the banner of decision theory. Decision theory concerns how agents reason or deliberate about making decisions [13]—concerns that are clearly relevant to morality. However, the question of what it means to make a decision, as opposed to how to tell if a certain decision is good or not, is only a recent focus for philosophy. As Jay Lampert states:

Decision only became a central topic in philosophy in the twentieth century. Before then, philosophy gave central roles to will and choice, to prediction and expectation, to imagination, desire, and hope. But decision has a different relation to the future than all of these [14 p. 2].

What’s unique about decisions is that they are future-oriented in a particular way. Further, their relation to the future is not always the same. To borrow one of Lampert’s examples, if I resolve to wake up at 8:00 AM tomorrow morning, it is clear what the temporal referent^5^ of that decision is: at a definite time tomorrow, I have decided to act in a certain way. However, what about more open-ended decisions? Lampert gestures towards resolving to try to combat climate change as a case of such a thing. For those in bioethics, one can imagine a patient resolving to be healthier, a doctor deciding to be nicer to her patients, or a chaplain deciding to live out her vocation. In these cases, it is clear that there are ways in which decisions can be lived out or fail to be lived out. However, the branching futures implied by such decisions are far from clear. After all, if I resolve to be nicer, that means I am resolving to act in many different ways that I cannot foresee in situations that are unknown to me. Should a stranger ask me for directions in an hour, though I would not have predicted this would happen, my past decision binds me to be nice and help this person.

Even a seemingly binary decision such as whether to withdraw life-sustaining care from a patient binds you to a complicated future. There are many different ways to enact that decision (Should the family be in the room? Should a chaplain? When should it be done? Should something be said during the moment before ‘pulling the plug’? What happens if someone bursts out crying?) and also many different sub-decisions that must be made along the way. In each case, whoever decides to withdraw life-sustaining care must reaffirm their decision by agreeing to act in accordance with it across the different sub-decisions across the different branching futures that confront the decision-maker. As Lampert says of complex decisions:

I look forward to a temporal succession in the real world that shifts just slightly act-by-act. Once I come to my decision’s second enactment, and its hundredth (obviously decision phases are not countable in this simple way), there will have been uncountable unforeseeable divergences. And yet this differential is what the decision’s forward-directed intentionality is about. The decision I make now is intended to affect an actual future, to treat actuality as a field of possibilities, and turn that possibility into a new actuality further in the future, which it expects to treat again as a possibility, and so on; but it does all this now, while possibility and actuality are both possible [14, p. 5].

When I decide, I am deciding because of and in light of future possibilities that will become actualities. Decision-making is necessarily futural in nature; one does not make decisions whose effects will realize in the past, or even the present. Hence, decision-making is likewise also an *imaginative* process. At least when my decisions are not completely unreflective nor wholly spontaneous, the future possibilities that I commit to when I make decisions are ones known to me through my imagining them.

## Shared decision-making, value, meaning, and ‘what matters’

What about SDM? Although SDM is a type of decision-making, the contours of mere decision-making do not exhaust the content of SDM itself. Russell Kirkscey provides a standard way to frame SDM:

Shared decision-making … entails a biomedical communication process between patient and provider that highlights patient autonomy, values, and treatment options informed by evidence-based medicine … with the goal of stakeholders’ mutual agreement [15, p. 1].

To extrapolate (using Kirkscey’s words but not necessarily his sentiment), SDM is frequently framed as a negotiation between the values of various stakeholders seeking some sort of agreement. This is meant to contrast with more traditional paternalistic models of decision-making in which a physician tells a patient what to do and the patient can either agree or refuse. SDM’s use of stakeholder analysis (hereafter ‘SHA’) reflects the general popularity of the method in bioethics, as well as in other healthcare field like health policy [16].

With the caveat that “stakeholder analysis means many things to different people” [17], SHA’s general method of operation involves assessing what stakeholders are relevant to the question at hand, assessing their values with regards possible outcomes, crafting a course of action which attempts to uphold as many stakeholder values as well as possible, and then iteratively checking in with stakeholders to see if they are still happy with what is being done or if their values have changed with regards to the process. One way of framing SDM is to frame it as a special case of SHA. In other words, SDM is SHA applied to clinical questions that affect multiple people.

However, this model alone is a deficient one. Authors in the SDM literature have noticed some important conceptual problems in these aspects of SHA, whether they’re specifically called out as aspects of SHA or not. Take, for example, Entwistle and Watt:

Reflecting [a need to look deeper at SDM], and recognizing that it is hard for people to have open discussions about what to do if they don’t have a shared understanding of the problem they are trying to address, we suggested that patients’ engagement in the recognition and clarification of problems should be considered an aspect of patient involvement in treatment decision making. This suggestion challenges the idea that judgements about problems are distinct from decisions about courses of action and that patient involvement is most important for decisions [18, p. 8].

A critical gap in SHA is that it takes the contours of the problem itself as granted. In other words, it assumes that all stakeholders perceive the state of affairs, the options, and the outcomes in roughly the same way as each other. What SHA hopes to facilitate is maximizing how many stakeholder values can be obtained in light of a common understanding of an issue.

I suggest that the problem goes even deeper. As distinguished from the quasi-naïve realism of SHA, SDM operates with the acknowledgement that the *meanings* of different events can differ among participants in decision-making (hereafter ‘participants’). Participants also may not count the same number of events nor understand the basic framing of the situation in the same way. Thus, SDM simply cannot be the mere consideration of different sets of values given the plurality of different possible meanings of different events. Indeed, there may be no basic agreement on even the basic frame of the issue by which a comparison of values can happen. What SDM calls for is the imagination of *meanings* present in the possible futures. In the spirit of this decision-making being *shared*, it is a *collective* exchange of imagination. That is to say, when I invite you to consider, I invite you to imagine.

Finally, the process of imagination involves asking not just about the meanings but which meanings *matter*. The gap between what a thing means and whether it matters is subtle, but important. If I hear 4 chimes while walking around outside, I am capable of discerning that this means it is 4 o’clock. However, that observation may not matter to me if it is a weekend and I am lazily passing the day away anyway. If I am in a hospital clinic and I see a heartrate monitor flatline, it means that the heart of the person in front of me has stopped. This could matter very much to me, especially if it is a person I know. Alternatively, if I am cold and detached from watching so many people die in the hospital, then it might not matter much to me at all.

And yet, one might object that this seems like a collapse back into the question of value once more. Does not one case matter and another not matter because of the values I hold? To show that no such collapse occurs, consider the case of a doctor who is trying to decorate their office. They put up various posters with motivational slogans on them, place some tasteful floral displays in the corner, and purchase new, comfy furniture to help visitors relax. Since their motivation is to make everyone feel at home, they decide to put up some religious symbols around the office. A Buddha head statue goes on a pedestal by the window, a painting of Muhammad reproduced from the Jami’ al-Tawarikh goes on a wall, and a cross of Saint Peter goes against another wall. Much to the surprise of the doctor, their first three patients of the next day, a Buddhist, a Muslim, and a Christian, are wildly offended by the décor and leave. The Buddhist finds the Buddha head offensive,^6^ the Muslim is angered at a pictorial depiction of the prophet,^7^ and the Christian perceives the cross of Saint Peter as a Satanic symbol.^8^ So, what happened? Was it the case that the three patients valued their religions whereas the doctor either disvalued them, did not value them, or rather valued blasphemy? It should be clear that this is not the case. Rather, what happened is that different things stood out as meaningful to the various parties and these meaning mattered to them. To say that the parties had differences of value is not accurate.

The future-oriented, imaginative exercise of SDM thus requires participants to engage in a mutual imagining of what is meaningful and what matters and to bring these things forward such that they can be deliberated upon. What is not clear from this account alone is precisely what to do once the various meanings that matter are brought together as a group. For instance, one would obviously suspect that some meanings that matter are more important to uphold than others (e.g. saving a patient who is screaming profanities in pain is far more important than requesting redress for being offended by being sworn at). Where SDM is not an exact, formulaic recipe for decision-making, the precise way to compare what matters to generate a decision is usually left under-specified.

This property is not unique to SDM. For instance, in the CASES approach^9^ to clinical ethics consultation, ethics consultation consists of five steps: clarify, assess, synthesize, explain, and support. Within the “synthesis” step, clinical ethicists are expected to “engage in ethical analysis” and “facilitate moral deliberation about ethically justifiable options” [19, p. 25]. However, there is certainly no concrete procedure given to carry out the process. As I said, this is not a lacuna unique to the CASES method, nor for SDM broadly. What I am trying to get at here is to say that all methods for decision-making in some way proceed from an assessment of the case to the generation of a course of action by means of some form of interchange/exchange about ethics. For the purposes of this paper, then, it is sufficient to say that, should the method for assessing a case be significantly different (e.g., by displacing a comparison of value in favor of a consideration of what is meaningful and what matters), then this should be a noteworthy difference in the general process of SDM—whatever analysis may come after the fact.

## Meaning in the *Umwelt*

If animals lack the cognitive or linguistic ability to engage in the imaginative and communicative demands of SDM, then it would seem that the project is doomed. One would have to either devolve back into a benevolent paternalism or an erasure, as I have intimated earlier. However, consider again the structure of SDM. What matters is based on what things mean to the participants in SDM. The trick is that what things mean is not a strictly linguistic question. Rather, the interpretation of signs as meaningful is a *semiotic* question, of which natural language is only one possible field. Much has started to emerge in the last 100 years or so of the importance of semiotic processes for biology. Semiotics itself has been present in Western thought since at least the Greeks. However, the importance of semiosis for biological processes is only comparatively recently gaining recognition. Jesper Hoffmeyer [20, 21] is a famous proponent of the relevance of biosemiotics, especially for biochemistry. Hoffmeyer and others (e.g. Sebeok, Emmeche, Favareau, Deely, Deacon, T. von Uexküll^10^ and many more) also see the application of semiotics to the biosphere, and especially animals, outright.

The modern origin of the study of semiotics in the animal world lies in the pioneering work of the Estonian-German biologist Jakob von Uexküll. Uexküll’s great contribution to philosophy and biology is his ‘discovery’ that animals do not simply reside in a prefabricated world, but rather that animals construct their environments according to their perceptions, interpretations, and actions. His term for the perceptual world in which an animal resides is the *Umwelt* (plural *Umwelten*) [8]. As Uexküll so frequently puts it, the *Umwelt* is like a soap bubble that represents the extent of what is present in an animal’s world. The best way to demonstrate this is with Uexküll’s own example of the sensory world of a tick. As Deleuze summarizes:

[Uexküll] will define this animal by three affects: the first has to do with light (climb to the top of a branch); the second is olfactive (let yourself fall onto the mammal that passes beneath the branch); and the third is thermal (seek the area without fur, the warmest spot). A world with only three affects, in the midst of all that goes on in the immense forest [22, pp. 124–125].

A tick perched on a branch can wait motionlessly for years at a time. It will remain perched there until it smells butyric acid (a compound of mammalian sweat), after which it drops onto its prey. It seeks a warm spot and feeds upon its first and final blood meal.

The other objects so familiar to the world of humans do not appear to the tick. The *Umwelt* represents not just what the animal ‘pays attention to,’ but indeed all that exists for the animal. Humans are no different. Against the backdrop of all possible sources of sensation, humans reside in their own *Umwelten.* Humans should not have the pretension that their world is the world as it truly is. This is a philosophical insight given to Uexküll most immediately from Kant.

What the *Umwelt* theory provides is an attempt to account for how the perceptions of animals have reciprocal relations with their surroundings. An animal is only capable of perceiving something that can make itself known to that animal’s particular sense organs—a concept that I have elsewhere dubbed “organ agreement.” If a human were to sit in a room being irradiated, though that would be a highly impactful event for that human, that person would not be able to perceive it happening. Similarly, beyond what *presents* itself to the animal, an animal’s perceptions *create* the *Umwelt* in which it resides. An animal’s horizon for experience exists precisely in this relational, mutually constitutive capacity.

The contents of the *Umwelt* are themselves carriers of meaning. Indeed, the key component of the *Umwelt* is precisely that its contents can be meaningful to the resident of that *Umwelt*. Against the backdrop of the general semiosphere—“i.e., the totality of actual or potential cues in the world” [20, p. 185]—the observer constructs for themselves a meaningful *Umwelt* according to the nature of their semiotic niche and their particular individual experiences. Within this *Umwelt*, the meanings of particular objects (recall that the objects that reside within the *Umwelt* are not pregiven) are liable to change as well. Take, for instance, the following example given by Uexküll:

An angry dog barks at me on a country road. In order to get rid of him, I grab a paving stone and chase the attacker away with a skillful throw… Neither the shape, nor the weight, nor the other physical properties of the stone have changed … and yet it has undergone a fundamental transformation: it has changed its *meaning.* As long as the stone was integrated into the country road, it served as a support for the hiker’s foot. Its meaning was in its participation in the function of the path … That changed fundamentally when I picked up the stone in order to throw it at the dog. The stone became a thrown projectile—a new meaning was impressed upon it. … The stone, which lies as a relationless object in the hand of the observer, becomes a carrier of meaning as soon as it enters into a relationship with a subject [8, p. 140].

The stone in and of itself is relationless. However, through entering into the Umwelt (that is to say, through its being *impressed*^11^ into an *Umwelt* by the perceptual act of the observer), the stone becomes a source of semiotic meaning.

## Animals and what matters

As I have claimed above, the experience of something as meaningful alone is not sufficient for that thing to be brought forth before others with forcefulness in SDM. What is needed, then, is to see if things *matter* for animals as well. Remember: not all meanings are relevant for the decision-making process. So, how, then, can one tell if something *meaning-fully matters* for animals? On one hand, consider Korsgaard’s analysis of the issue:

An animal is an organism that functions, at least in part, by representing her environment to herself, through her senses, and then acting on those representations. She is guided by her representations to get the things that are good for her avoid the things that are bad for her, in the functional senses of good-for and bad-for. In order for an animal’s representational system to do its work in this way, however, it has to have what I will call a “valenced” character. That means that the things she encounters in her environment have to strike her as attractive or aversive, welcome or unwelcome, pleasant or painful, in particular ways, depending on whether and how they are good- or bad-for her. She has to be drawn by the way things appear to her to seek out the things that are good-for her and to avoid the things that are bad. So she has to perceive the world evaluatively, as a place full of things which present themselves as attractive and to-be-sought and things which present themselves as aversive and to-be-avoided [24, p. 20].

Korsgaard, though not directly evoking language of semiotics, nevertheless establishes that the valenced character of animal perception that evaluates what an animal perceives is an important aspect of how an animal engages with the world. The valenced character of how an animal thereby engages with the world is an instance in which something can *matter* for an animal.

Korsgaard’s language of valence is shared among other scholars. Indeed, her observation that (at least many) animals experience the world with affective valence is a mainstream belief within the realm of animal welfare science. The premise here is to defer focus on particular, discrete emotions in favor of characterizing the core affect of a sensation by charting it along the axes of valence and arousal [25]. So, rather than say that an animal is joyful, one might say that it demonstrates an affect with positive valence and high arousal. Likewise, annoyance is thereby an affect with negative valence and low arousal. One of the promising features of using this framework is that one might use it to avoid anthropomorphism in the attribution of emotion to animals. While a given animal may certainly *seem* annoyed, how can one be sure that the animal perceives its emotional state as annoyance? The wager is that it is easier to observe valence rather than identify a particular emotion.^12^ Dawkins [27] sees the affective state framework as a possible expression of half of her general theory of welfare, which is a state where animals are healthy and have what they want.

So, regardless of the nuances, the philosophers and the scientists here have some agreement on the language to isolate at least part of what it would mean for something to matter for an animal. However, one must also see whether or not what matters also has *meaning*. For this, I look to John Deely, who writes:

The organism does not simply respond to or act in terms of what it senses as sensed, but rather in terms of what it *makes* of that sensation, what it perceives to be sensed, rightly or wrongly. The female wolf responds to the male’s howl differently than does the sheep, regardless of gender. Thus, whereas sensation prescissed and taken as such actively filters but passively receives incoming stimuli, perception by contrast actively structures sensation into things to be sought, things to be avoided, and things that don’t matter one way or the other. Yet what constitutes a pattern of stimuli as desirable and to be sought or menacing and to be avoided depends less on the stimuli than upon the biological constitution of the organism receiving the stimuli. Thus, the pattern of stimuli, in perception as contrasted to sensation as such, is actively woven, not passively received. Between and among sensory elements of stimulation, the organism itself weaves a network of subsequent relations which obtain only in the perceiving, not prior to and independent of it. It is the pattern of this network of relations within perception, not any prior pattern within sensation alone, that determines and constitutes the objects of experience so far as they are distributed into the categories of desirable (+), undesirable (–), and neutral (¸). Perception does no more [28, p. 128].

Deely, like Korsgaard, gestures towards the valenced nature of animal perception. Note that Deely, like Korsgaard, points out the gap between mere sensation as such (whatever that may mean) and the perceptual world of the animal. Korsgaard refers to this as the animal’s representation of its environment to itself whereas Deely refers to this as perception itself. The terminological differences are not in themselves all that noteworthy in that Korsgaard and Deely were not writing with each other’s terminology in mind, but the effective upshot of Deely’s comments is important: it is through semiotic processes that an animal inhabits a valenced perceptual space.

As a final step, it is important to ask to what extent this semiotic process requires the cognitive resources that animals may possibly lack. To look to Deely one last time:

Each species constructs and lives within *its own* lifeworld. The whole process is executed by means of signs, but the perceiving organism does not think of the matter in that way. It simply uses signs, as Maritain best put it, without realizing for a moment that there are signs. For whenever one element of experience makes present something besides itself, be that other ‘real’ or not (for example, the danger perceived only through an erroneous amplification of the stimuli of sense), the element in question is functioning as a vehicle of signification. This is why Sebeok so aptly speaks of experience as ‘a semiotic web’, that is to say, a web woven of sign relations, at whose nodes alone stand the objects of experience as experienced, whatever be their further status as ‘physical’ or ‘real’ independently of the experience within which they are given [28, p. 128].

This is the crux of this argument. Semiotic residence in an *Umwelt* allows for the existence of semiotically-meaningful, valenced perceptions *regardless* of whether one is a cognitively-linguistically savvy subject.

## Being an imaginative adjunct

Animals are therefore capable of experiencing meanings that matter even if they cannot necessarily manipulate these meanings in a way that reflects linguistic aptitude.^13^ The final step for SDM with animals, then, is to figure out what it would mean to allow what matters to animals to be present before a group of decision-makers. Even though animals have things that matter to them, their lack of linguistic skill means that they cannot *directly* engage with the communicative, imaginative demands of SDM. Imaginative, hypothetical scenarios cannot be shared with them through language, and they cannot speak the results of their imagining back with a human interlocutor. However, where SDM is not a philosophical dialectic, it does not require meaning to be shared only after being grounded in some further reasoned justification. So, for instance, one can share a desire that arises from what is proper to one’s culture without having to provide further reasoning about why cultural beliefs ought to be tolerated, without needing to provide a metaphysical ground for the particular beliefs, etc. What to *do* about the expressed beliefs is another question, but within SDM these sorts of things may be shared and ought to be taken seriously regardless of further philosophical deliberation.

Because SDM does not simply endeavor to find what values currently exist among stakeholders, the fact that animals may or may not have a list of propositionally statable values is not fatal. Rather, to the door is open to investigate what matters to animals in the imagined futures relevant to the process of SDM at hand. The tense of SDM is not ‘what matters to you,’ but ‘what would matter to you if…’ This question, while not directly answerable by an animal itself, is nevertheless one with a conceivable answer. Unlike propositionally statable values which may or may not exist depending on how the science surrounding animal cognition shakes out, one can be more confident that animals have things that matter to them owing to their residence in a *Umwelten* with meanings that matter to them within it. Hence, the way in which animals can participate in SDM is through having someone try to imagine on behalf of that animal what matters in such-and-such a future.

With regard to supported decision-making, several authors [29, 30] observe that the process can involve trusted people who represent a benefactor (e.g., a patient) in the process of decision-making. Despite the fact that a person or group of people is helping decide on behalf of the benefactor, supported decision-making is emphatically not a guardianship process. In the portrait I have developed here, a similar idea applies to animals in SDM. Imaginative adjuncting is a way to aid what matters to animals in being presented before a group for decision-making. Hence, it is a way to think of what it means to make shared decisions with animals and avoid both mere paternalism and erasure.

## Two test cases: shared decision-making in the laboratory and at the zoo

I want to conclude this paper by looking at what this theory of SDM would have to say about some concrete cases. The frame I’ve developed here is both a means to make shared decisions with animals as well as an analysis tool to understand whether a given decision is shared or not.

The first case (the simpler of the two) involves laboratory experimentation on rats. Empirically, rats are distressed in a significant proportion of experimentation, whether because of handling, direct pain inflicted, or whatever it may be. Further, most all rats involved in experimentation will be euthanized after their use in an experiment. Moreover, many ‘surplus’ rats (e.g. those bred but not needed, those who do not exhibit desirable traits for experimentation, etc.) are euthanized before ever even being used in an experiment [31].

Consider a hypothetical. Despite the obvious harms involved in animal experimentation, the defenders of the practice believe it is justified because of the benefits the experimentation generates. To provide informed consent to non-therapeutic research typically entails understanding that the harms a participant will suffer will be for the purpose of benefitting others. This would suggest at least some degree of altruistic motivation to participate without ulterior motive. There is in fact support for altruism in rats, at least at the behavioral level [32]. To recontextualize a point made by Wendler and Shah [33] in the context of pediatric research participants: would the fact that rats seem to possess altruistic values^14^ make a difference for whether one should consider them willing participants in biomedical research?^15^ Said another way, if rats’ values accord with self-sacrifice for the benefit of another, would one be helping them achieve their values by enrolling them in some research trials?

The analysis I’ve given accounts for why this is not really a case of SDM. While one could argue that it is in a rat’s *best interests* to participate in research (given their values),^16^ nevertheless, nothing that takes place in the context of a research trial would *mean* to a rat that it has helped others—except, perhaps, in those trials specifically designed to imperil a rat and then let another come to its aid. A rat would not believe at the conclusion of a trial that it has helped another rat, owing to no salient perception that would mean that it had done so. Thus, this would not be a case of SDM.

For a more complicated case, consider animals in zoos. Heini Hediger is widely recognized as inaugurating the investigation of semiotics (or semiotics-adjacent material) in the context of zoo animals. Turovski [34] claims that the theoretical basis for Hediger’s observations themselves derive from Uexküllian *Umwelt* theory. For instance, Hediger [35, 36] notes the importance of whether animals view their keepers with indifference, as friendly non-conspecifics, as inanimate environmental features, etc., as well as the perceptual differences between an animal’s home range, unfamiliar environments, nests, roosts, and so forth. These perceptual categories are themselves important drivers of animal behavior as well.

Take, for instance, primates’ perceptions of their own territory. In a zoo, sometimes captive primates will make aggressive displays towards visitors. As Hosey notes:

In the case of aggressive interactions with visitors, it is instructive to look more closely at the housing of the animals involved. In Wormell et al.'s study, two separate groups of tamarins showed aggressive behaviors toward the public, but by far the most threats (7.63 per animal per hour) were shown by a group in an enclosure with a high visitor number, with markedly less (0.06 per animal per hour) in the second group in which visitor attendance was lower. The authors interpreted this aggression as territorial defense [37].

Hosey himself goes on to give further analysis of visitor-directed and keeper-directed aggression in primates. However, even with this observation alone, there is something interesting to consider. What status does territorial integrity have for these tamarins? What is noteworthy that tamarins are capable of perceiving certain things in their environment (e.g., repeated presence of visitors) as *meaning* that their territory is threatened.

Consider, however, that the meaning they perceive is not exhausted by the claim that they value the protection of their territory. After all, their territory is factually not threatened by visitors who are kept outside the enclosure by the very walls that keep the primates in. Yet, it is not right to say that, when considering the interests of the tamarin, that they are fully taken care of just because their territory is not *actually* threatened by visitors. Rather, to represent the interests of tamarins, one must account for the fact that certain displays, behaviors, events, etc., *mean* that their territory is compromised, and that this *matters* to them. In the case of some sort of SDM process that would unfold involving the tamarins, the others involved in the process must consider these meanings.

Two objections come to mind: (1) that this is actually just a welfare concern masquerading as something more sophisticated (e.g. that all that matters is whether the tamarin is stressed, not what it thinks is going on), and (2) that this relativizes value/meaning to a deleterious degree. Although conversations about those questions could stretch on into another paper, I want to at least give them initial answers.

As to the first objection, the point is not directly answerable outside of reference to a more fundamental theory of value. If the only truly existing normative values are welfare, then trivially all that really matters is welfare. However, in other cases, I do not think that the portrait I have painted reduces to welfare concerns. Although it is true that animals tend to exhibit less stress when they obtain the things they want and when things that matter to them are taken into consideration, the same could be said of humans as well. When humans get what they want, they tend to be satisfied, and when they do not get what they want, they tend to be frustrated. That welfare concerns are *attendant* to the decision-making process does not illustrate that they are *exhaustive* of the decision-making process.

For the second objection, I would suggest that value/mattering are already partially relativized in ordinary clinical decision-making. Patients are free to suggest that they have cultural beliefs that matter to themselves and their care team is expected to engage with that value even if they do not share the same culture as their patient. This works even when those beliefs have reference to more fundamental values. For instance, an American woman may be shocked upon visiting a European OB/GYN where it is atypical to give a woman a disposable gown to wear during examination. An American woman, used to receiving one, may feel as though the nakedness offends her dignity. Is this simply an irrelevant, culturally-relativized belief? I do not believe it to be—nor would this belief deleteriously relativize the concept of dignity itself. Rather, it seems that a doctor should consider that nakedness might matter to this patient in a way that does not for other patients. If that is the case, then I argue that there are not problems when applying this standard to decision-making with animals either.

## Conclusion: the need for a critical cognitive ethology

Shared decision-making, then, demands the task of *imagining on behalf* of animals. To take their interests seriously requires not only taking the values of other animals seriously, but also their meanings, which in turn requires the attempt to enter into animals’ *Umwelten.* Such a task is certainly not an easy one. Projecting too strongly onto animals risks anthropomorphism. However, what the process aims at is not false projection, but rather attempting to translate what matters for animals in a way that is understandable for humans. The process will inevitably end up producing chains of equivocation whereby one and the same thing might mean different things to different participants, as no one *Umwelt* represents the correct interpretation of the semiosphere. Commenting on the question of anthropological comparison, Viveiros de Castro writes:

Anthropology, then, is interested in equivocations in the ‘literal’ sense: *inter esse,* betweenness, existing among. But, as Roy Wagner said of his initial time with the Daribi of New Guinea, ‘their misunderstanding of me was not the same as my misunderstanding of them,” (which may very well be the best definition of culture ever proposed). The critical point, of course, is not the mere fact that there were empirical misunderstandings, but the ‘transcendental fact’ that they were not the same. The question, accordingly, is not who was wrong and still less who misled whom. Equivocation is not error, deception, or falsehood, but the very foundation of the relation implicating it, which is always a relation with exteriority. Deception or error, rather, can be defined as something peculiar to a particular language game, while equivocation is what happens in the interval between language games [38, p. 40].

So too is it across species. Humans’ misunderstanding of animals is not the same as animals’ misunderstanding of humans. Between the two lie equivocal perceptions of the world. To pursue the perceptions of the other, SDM with animals calls for the serious investigation of critical cognitive ethology and its implications for the clinic. I do not claim, of course, that figuring out what a given thing will mean to a given animal is an easy task—far from it. If one could simply pluck the contents of an animal’s perceptions from their mind, then interaction with animals would be made all the easier. But, alas, this is not our lot.

What remains, then, is investigatory and open-ended. Will the separation of a dog from its owner mean social abandonment to the dog, the inability of the dog to protect its owner, or will it remain indifferent? How does a cat experience being kept in a clinic for several days? These are questions which do not admit of convenient answers, either at the level of species or for an individual. However, I hope my account has demonstrated the use in pursuing such questions. The ethos of shared decision-making is one which belongs to human-animal encounters as well.

## Footnotes

1 I ask the reader’s forgiveness for having to qualify so many terms like this. The fact of the matter is that there is still much that is murky in this problem area. Language is very complicated, cognition is very mysterious, and the animal kingdom is very broad.

2 In brief, semiotics is the study of signs (e.g., the relationship between carriers of meaning and meaning itself).

3 In brief, an *Umwelt* is the perceptual world inhabited by an organism owing to its own sensory apparatuses.

4 Simmons and Gooding [10] say this when analyzing *supported* decision-making. However, they see shared and supported decision-making as sharing many more similarities than differences. Owing to the closeness of the two categories, I see it fair to fit the quote in here as well.

5 Lampert himself refers to “the temporal character of the decision object … [as] the temporal noema of the decision” [14, p. 3].

6 Some Buddhists perceive that Buddha heads resemble trophies taken by colonizers.

7 Many Muslims believe that all visual depictions of the prophet are strictly forbidden.

8 Although a traditional symbol of piety, the inverted cross of Saint Peter has also been used as a symbol by Satanists.

9 CASES is the general model for ethics consultation used in the USA Veterans Affairs’ healthcare system.

10 Not to be confused with J. von Uexküll, whom I will mention shortly.

11 Space does not permit me to talk of the importance of the term *hinausverlegt* for Uexküll’s thought. For more, see Hoffmeyer [23].

12 While I have introduced the affect framework here as a curative for some anthropocentric bias, some risks for anthropocentrism may remain. See [26].

13 Again, they might be able to do this after all. However, where the current state of the evidence is so divided, I am remaining agnostic on the fact.

14 A leap from the data, but not an unreasonable one per se.

15 To be clear, Wendler and Shah believe that one should look for not just *implicit* altruism in pediatric patients, but rather a more full-bodied appreciation of how their altruism would relate to a proposed research trial.

16 I am certainly skeptical that it is really in their best interests, to say the least of the matter.
